# The Future of Coaching: A Conceptual Framework for the Coaching Sector From Personal Craft to Scientific Process and the Implications for Practice and Research

**DOI:** 10.3389/fpsyg.2021.715228

**Published:** 2021-11-10

**Authors:** Jonathan Passmore, Rosie Evans-Krimme

**Affiliations:** ^1^CoachHub GmbH, Berlin, Germany; ^2^Henley Business School, University of Reading, Reading, United Kingdom

**Keywords:** positive psychology, coaching research, coaching practice, democratisation of coaching, coaching trends

## Abstract

This conceptual paper explores the development of coaching, as an expression of applied positive psychology. It argues that coaching is a positive psychology dialogue which has probably existed since the emergence of sophisticated forms of language, but only in the past few 1000years, has evidence emerged of its use as a deliberate practice to enhance learning. In the past 50years, this dialectic tool has been professionalised, through the emergence of professional bodies, and the introduction of formal training and certification. In considering the development of the coaching industry, we have used Rostow’s model of sector development to reflect on future possible pathways and the changes in the coaching industry with the clothing sector, to understand possible futures. We have offered a five-stage model to conceptualise this pathway of development. Using this insight, we have further reviewed past research and predicted future pathways for coaching research, based on a new ten-phase model of coaching research.

## Introduction

Coaching is often considered an applied aspect of positive psychology. Both emerged from humanistic psychology, with its focus on the flourishing of the individual, and how individuals, teams and society can create the right conditions for this to be achieved. In this paper, we explore the nature of coaching, as an applied aspect of positive psychology, the journey so far and where practice and research may be heading over the coming 30years.

It would seem only prudent at the start of this paper that we note the challenges of predicting the future direction of any industry and of research in general. We acknowledge the future is ‘trumpet-shaped’, emerging from the point of singularity (now) to multiple possible futures. Any attempt to accurately ‘predict’ the future is challenged by inevitable unforeseen events, their timing and the interaction between foreseen and unforeseen events. We have tried to improve our predictions by drawing on a previously published framework, which we have adapted. However, we ask the reader to note this is just one possible future.

## What Is Coaching?

The clear link between coaching as lived positive psychology has been focused on by many writers (Lomas et al., 2014). However, just how much of a driving force positive psychology was in the maturation of coaching is yet to be discussed. In order to establish the role positive psychology played in the maturation of the coaching sector, this section will review their shared historical roots and focus on the influence positive psychology research had on the evolution of coaching definitions.

Coaching and positive psychology’s histories are dynamic and rooted across multiple disciplines, yet they are both born out of the Human Potential Movement of the 1960s. This led to the popularisation of personal and professional growth and development, through pioneers such as Werber Erhard. Brock’s (2010) review of the development of coaching notes the humanistic tradition and the work of Carl Rogers’ of particular significance. Rogers’ focus on the relation and the needs of the clients’ and the potential to find their own way forward have become central features of coaching. Coaching rise coincided with a shift in the perspective about illness and wellbeing. This was the move from the medical model that focused on pathologies to the wellbeing model, which encouraged greater attention towards what individual’s strengths.

Interestingly, coaching psychology (CP) launched in the same year as positive psychology. Atad and Grant (2021) described how coaching psychology was a ‘grassroots’ movement, led by founders of the Coaching Psychology Unit at the University of Sydney and the Special Interest Group in Coaching Psychology in the British Psychological Society (BPS) including psychologists like Stephen Palmer, Jonathan Passmore and Alison Whybrow.

While positive psychology and coaching psychology both focus on the cultivation of optimal functioning and wellbeing (Green and Palmer, 2019), often through the use of personal strengths development, Atad and Grant (2021) reported key differences. The first is that approaches like solutions-focused cognitive-behavioural coaching also aim to help clients define and attain practical solutions to problems. A second difference is the characteristics of the interventions used in each discipline. In coaching psychology interventions, the coach-coachee relationship is central to the coachees development of self-regulated change, whereas Positive Psychology Interventions (PPI’s) typically apply a self-help format.

Since its foundation, positive psychology (PP), the ‘*scientific study of positive human functioning and flourishing intra-personally* (e.g.*, biologically, emotionally, cognitively*)*, inter-personally* (e.g.*, relationally*)*, and collectively* (e.g.*, institutionally, culturally, and globally*)’ (Seligman and Csikszentmihalyi, 2000; Oades et al., 2017), has grown into a field of applied science (Atad and Grant, 2021).

The field covers an array of topics, most commonly focused on life satisfaction, happiness, motivation, achievement, optimism and organisational citizenship and fairness (Rusk and Water, 2013). Gable and Haidt (2005, p. 103) defined positive psychology as ‘*The study of the conditions and processes that contribute to the flourishing* (*wellbeing*) *or optimal functioning of people, groups, and institutions*’. A vast number of PPI’s have been developed and validated (Donaldson et al., 2014), with the aim to enhance subjective or psychological wellbeing or to cultivate positive feelings, behaviours, or cognitions (Sin and Lyubomirsky, 2009).

Strength development is viewed as a key process in positive psychology due to the shift from a deficit-based approach towards human functioning: a move from ‘what’s wrong and how can we fix it’, to ‘what’s right and how can we strengthen it’. McQuaid (2017, p. 285–284) simply describes strengths as, ‘*the things you are good at and enjoy doing*’, which is reflective of Linley and Harrington’s (2006) definition of strengths as ‘*a natural capacity for behaving, thinking, or feeling in a way that allows optimal functioning and performance in the pursuit of valued outcomes*’. Strength-based interventions that aim to promote the awareness, cultivation and application of personal strengths have been reported to have many positive individual and organisational outcomes (McQuaid, 2017). Strengths research is one of the most integrated concepts from positive psychology into coaching psychology.

Interestingly, coaching psychology (CP) also launched in the same year as positive psychology. Atad and Grant (2021) described how coaching psychology was a ‘grassroots’ movement, led by founders of the Coaching Psychology Unit at the University of Sydney and the Special Interest Group in Coaching Psychology in the BPS in the United Kingdom. CP also experienced its own rapid growth in research and practice (Green and Palmer, 2019), which is discussed below.

At the heart of coaching, noted by multiple coaching writers, was its facilitative nature (Passmore and Lai, 2019). Coaching pioneer John Whitmore’s working with Graham Alexander and Alan Fine in the later 1970s and 1980s, and informed by the work of Tim Gallwey (1986), focused on the self-awareness and personal responsibility which coaching created. This led to Whitmore (1992) defining coaching as having the potential to maximise a person’s performance by adopting a facilitation approach to learning rather than teaching.

This has direct parallels with Deci and Ryan’s (1985) work on self-determination theory (SDT). SDT identifies the conditions that elicit and sustain motivation, focusing on self-regulated intrinsic motivation. These include the needs for competence, autonomy and relatedness (Deci and Ryan, 1985). While Whitmore never formally engaged with SDT Whitmore’s definition of coaching can be seen as a direct application of Deci and Ryan’s (1985) SDT theory and therefore an early indicator of coaching as an applied aspect of positive psychology.

In Brock’s (2010) reflection on the common themes, the facilitative nature of coaching is the strongest similarity across definitions. Brock (2010) also emphasised the interpersonal interactive process that places the coaching relationship at the centre of the facilitation and essential for positive behavioural change. This perspective was maintained in later definitions, which introduced the purpose of coaching to drive positive behavioural changes (Passmore and Lai, 2019) For example, Lai (2014) reported that this was driven by the reflective process between coaches and coachees and continuous dialogue and negotiations that aimed to help coachees’ achieve personal or professional goals.

In summary, in spite of the complementary nature of PP and CP, the fields remain sisters as opposed to fully integrated areas of practice, with coaching psychology drawing from the well of positive psychology, alongside wells of neuroscience and industrial and organisational psychology.

## A Conceptual Model for Coaching Development

To date, little has been written about the development of the coaching sector, as a specific industry. This may reflect in part the relative immaturity of the industry, but a wider review of industrial literature reveals the categorisation of sector development is limited. One of the few conceptual models is Rostow’s (1959) generalised model of the six stages of economic growth. This offered a linear model of development, which reviews traditional society; the preconditions for take-off; the take-off; the drive to maturity; the age of high mass consumption; and beyond consumption (the search for quality). The model is summarised in Table 1.

**Table 1:** Rostow’s stages of economic growth.

Stage		Key characteristics	Key manifestations
1	The traditional society	Subsistence economy Primary sector economy No surplus Lacks modern technology Poor economic mobility No centralised system for growth	Small technological advancements that improve processes
2	The preconditions for take-off	Shift towards an industrial society: modern alternatives to traditional approaches Advancements driven by technology and science Wholly primary sector economy	Increased demand for key industries or sectors Improvement of conditions for productivity and trade, including investment. Creation of industrial markets Reduction of waste surplus
3	The take-off	Rapid self-sustained growth Secondary economy expands Industrialisation continues to dominate Urbanisation increases	Growth limited to key sectors Sectors become resilient
4	The drive to maturity	Diversification of sector development Shift to consumer-driven investment	Acceleration of new sectors and deceleration of old sectors Significant investment into transportation and social infrastructures
5	The age of mass consumption	Enabling of widespread consumption of consumer goods Expansion of tertiary sector	Increase in urbanisation Hypergrowth across secondary and tertiary sectors Population has growing disposable income (Increased inequalities between high and low socio-economic countries) Application of scientific insights and research
6	Beyond consumption (the search for quality)	Impact of high consumption changes consumer behaviour/mindset: seeks for durability and sustainability	Resources are draining Natural and man-made disasters having an impact of the economy

Rostow argued that these stages captured the dynamic nature of economic growth, reflecting the nature of consumption, saving, investment and the social trends that impact it. Rostow’s framework offered insight into the triggers for change at each stage. However, as a generalised model, it is unlikely that all industries or sectors follow the same pathway. Some may never take-off, and others remain as mass consumption. It is also important to note that Rostow’s model is based on observations from a predominantly Western economic perspective situated within a capitalist economic model of growth.

We believe this model provides a heuristic guide, a map, for those observing the development of coaching, and offers an opportunity to predict, based on trends in other industries, how coaching may develop over the coming few decades. In undertaking an analysis of the model, it may be helpful to explore it through a specific sector. The example we selected was clothing manufacture, as being a process which like language, dates back to the prehistory, but which has also changed and developed over the centuries.

## The Clothing Sector

The clothing sector has undergone a transformation over the past 10,000years. We might start by considering ‘the sector’ at the time of the Neolithic Revolution, as humans transitioned from hunter gathers to farmers. In hunter-gatherer societies, clothing was primarily a form of protection: protection from cold, plants, animals and battles with fellow tribes. Although given evidence from modern day hunter-gatherer societies, there are also limited examples of parts of clothing being used for status, for example Native American head-wear (Grinnell, 2008). As humans settled, this too started evolve with the emergence of greater status divisions and the development in manufacturing of items, allowing greater differentiation of objects. In the earliest period, most people will have collected the raw materials, engaging as a group in killing an animal. They will have prepared the materials in small groups, stripping the flesh and processing the hide and finished the item will individuals sewing to weaving items together to form the clothing.

As food surpluses emerged as a result of the shift towards settled farming, specialisms started to also emerge. Clothing production shifted from the collective task for small groups and individuals, to the one or more specialists, such as a tailor. This process of specialisation continued with the emergence of training and the development of trades: where individuals could progress over several years of training form apprentice through journeyman to master craftsman. Alongside, this came trade bodies and guilds in the 12th and 13th centuries, to represent the profession and to protect members rights (Ogilvie, 2011). The industrial revolution brought further change with production moving from cottage industries, small shops or upstairs of building used as part home and part clothing ‘factory’ to formal factory production using mechanisation to increase consistency and reduce costs. This process has continued with continued development of automation and over the past 30 years through the digital revolution, which has witnessed a shift from individual’s controlling machines to machines controlling machines. Table 2 summarises the transformation of the clothing sector and demonstrates how Rostow’s model could be applied.

**Table 2:** Model of clothing sector development based on Rostow’s model.

Stage		Key characteristics	Key manifestations
1	The traditional society	Subsistence lifestyle Need for survival (stay warm and dry)	Humans wrapped furs, leathers, and plants around their bodies
2	The preconditions for take-off	Specialisation revolution Need for trade Cottage industry Labour intensive	Foundational technology for modern day clothing production was discovered, basic sewing needles Master craftsmen specialised in tailoring, which enabled trade
3	The take-off	Labour revolution Need to productivity Basic clothing production practices developed and defined Trading of materials and clothing Clothing manufacturing as a personal craft	Tailors master their craft Workers employed to produce materials for textiles and clothing Technological innovations increased productivity and efficiency of producing key textiles, such as cotton Surplus materials and clothing could be traded.
4	The drive to maturity	Industrial revolution Shift from craft into a service Automated production Need for expansion Clothing manufacturing as a technical process	Application of technology to transform textiles into mass-produced, consumer goods Factories specialised in production and manufacturing of certain products and clothing Master craftsmen (i.e., designers) become highly specialised and desired Emergence of sector bodies to represent the profession and protect members’ rights, guilds, and trade bodies
5	The age of mass consumption	Diversification revolution Shift to consumer-driven market Need to reduce production costs and increase profits	Globalisation increased trade and exchange of clothing goods Increased accessibility of lower labour and production costs, often due to automation of machinery and processes Population has a growing disposable income and consumers demand access to fashion items Increased availability of high street and cheap, fast-fashion brands; clothing not designed for life-time use.
6	Beyond consumption (the search for quality)	Decline and fall: impact of sector practices and society on sector, people, and environment Clothing production and manufacturing as human rights and climate emergency	Human rights violations against factory workers in low-economic countries in order to meet demands from consumers in high-economic countries Rise in sustainable fashion practices to negate the impact clothing production and throw-away society has on the environment

## Coaching: The Future of Research and Practice

We argue that coaching can learn from the evolution of these other sectors and from the wider conceptual model proposed by Rostow, to better understand the future direction of the coaching and its implications for practice and research.

We start by suggesting that coaching is likely to have a prehistory past. While some argue that coaching was born in 1974 (Carter-Scott, 2010), we believe it is almost certain hunter gathers will have engaged in the use of listening, questioning and encouraging reflective practice to help fellow members of their tribe to improve their hunting skills or their sewing. There is some evidence from Maori people, in New Zealand, that such questioning styles have been used for centuries to aid learning (Stewart, 2020). However, the spoken word leaves no trace for archaeologists to confirm the development of these practices.

While the clothing sector developed in full sight, leaving traces for archaeologists in graves and wall paintings, coaching remained a hidden communication form, until its emergence in societies where written records documented different forms of learning. At that moment, the Socratic form was born. It is often this moment which until now has been regarded as the birth of the positive psychology practice of coaching. It has taken a further 2,500years for coaching to move from a learning technique used by teachers to a specialisation increasingly concentrated in the hands of the few, which requires training, credentials, supervision and ongoing membership of a professional body. While there is good evidence of individuals using coaching in the 1910s (Trueblood, 1911), 1920’s (Huston, 1924; Griffith, 1926) and 1930’s (Gordy, 1937; Bigelow, 1938), the journey of professionalisation started during the 1980s and 1990s, with the emergence of formal coach training programmes and the formation of professional bodies, such as the European Mentoring and Coaching Council in 1992 and International Coaching Federation in 1995. The trigger for this change is difficult to exactly identify, but the growth of the human potential movement during the 1960s and 1970s and its focus on self-actualisation, combined with the growing wealth held by organisations and individuals meant a demand for such ‘services’ started to emerge from managers and leaders as part of the wider trends in professional development which started in the 1980’s.

This trend of professionalisation has continued for the last three decade. The number of coaches has grown to exceed some 70,000 individuals who are members of professional bodies and industry, although given data from recent studies which reveal that over 30% of coaches have no affiliations, we estimate over 100,000 people earn some or all of their income from coaching (Passmore, 2021). In terms of scale, the industry is estimated to be worth $2.849 billion U.S. dollars (International Coaching Federation, 2020), but in many respects, it has remained a cottage industry, dominated by sole traders and small collectives, with little consolidation of services by larger providers, with little use of technology and science to drive efficiencies or improve outcomes.

Given model and recent developments in technology and the growth of coaching science over the past 10years is coaching reaching a tipping point? Is coaching about to enter the next phase of sector development? Is coaching about to begin the transition from professional service delivered by a limited number of high-cost specialists to an industrial process capable of being delivering low-cost coaching for the many with higher standards in product (service) consistency?

What makes this change likely? There are three factors in our view propelling coaching towards its next stage in development. Firstly, the growth of online communications platforms, such as Microsoft Teams, Zoom and Google Hangout, are enabling individuals to connect with high-quality audio and video images. The impact of the COVID-19 pandemic during 2020–21 has seen the development these platforms now reach almost universal adoption. At the same time, a growing number of employees have switched from ‘always in the office’ modes of working to either working from home or hybrid working, working 2, 3 or 4days a week from home (Owen, 2021). Such models provide lower costs for employers, and evidence suggests many employees favour the flexibility working from home provides.

Secondly, the period 2010–2020 witnessed a growth in the science connected with positive psychology and coaching, proving practitioners with a good understanding of the theory and research. Access to this research has been enhanced by an increasing move to Open Access journals, the emergence of research platforms, such as ResearchGate, sharing published papers and tools such as Sci-Hub, granting access to published science, alongside search tools such as Google Scholar allowing efficient discovery of relevant material by practitioners, as well as academics with access to university library databases. In combination, these online tools are democratising the science of coaching and are stimulating the next phase of development.

The third factor is the growth of investor interest in digital platforms, which have seen significant growth during the 2010–2020 period, enabling start-ups to secure the investment need for the development of products, from online mental health (Headspace) to online learning (Lyra Learning - LinkedIn Learning).

The next phase we predict will be an emergence, growth and ultimately domination of coaching by online large-scale platforms, who offer low-cost and on-demand access to coaching services informed by science, in multiple languages and to a consistently high-quality standard. Echoing the changes in clothing production, with mechanisation using machines like Eli Whitney’s cotton gin, and Arkwright’s spinning machine which revolutionised clothing production.

We have developed Rostow’s model and propose a 5P’s model for the coaching industry development. This is summarised in Table 3. A journey from unconscious practice used by hunter-gatherer societies, through formal use in learning, to specialisation and professionalisation, to the deployment of technology and onwards towards a more conscious use across society of positive psychology approaches, including coaching as a tool to enhance self-awareness and self-responsibility, embedded in technology.

**Table 3:** 5P’s model of coaching industry development.

Stages	Characteristics	Change
Stage 1: Peoplisation: (50,0000–5,000years ago)	Coaching as unconscious conversational tool, part of daily dialogue:	Coaching emerges as part of sophisticated language
Stage 2: Purposisation: (5000–50years ago)	Coaching with explicit learning goals	Coaching adopted by specialists, such as Greek Philosophers and others, to enhance learning
Stage 3: Professionalisation: (50years ago to today)	Specialist coach training, standards and certification	Emergence of professional bodies setting standard, training and accreditation leading to the creation of a profession
Stage 4: Productisation: (Mid 2020s onwards)	Coaching with science and technology	Emergence of specialist companies combining technology and science to offer lower cost, consistent and high-quality coaching process and outcomes
Stage 5: Popularisation	Many streams of coaching emerge	Coaching continues as a niche upmarket service by professionals, as an industrial process for the many at work and consciously adopted for use in personal encounters as part of daily dialogue for all encounters embedded in technology

## Risks

It is worth noting that across sectors the move from one stage to the next created disruption and negative consequences in uncontrolled markets. In agriculture, shifts in production, such as land enclosures, and introduction of mechanisation led to landlessness and starvation, in clothing manufacturing production disruptions lead to low pay and exploitation. These changes also stimulated agricultural revolts and the emergence of Luddites, as workers affected by change pushed back against these change in their daily work patterns, income levels or status.

In coaching, we can see similar push back from some in coaching, who fear the negative impacts of research and technology, as coaching starts to move away from being a cottage industry, where fee rates are unrelated to training, qualifications or other measurable indicators (Passmore et al., 2017) towards providing greater consistency, evidence driven practice. Such push back is likely not only to be from individuals but also guilds (professional bodies) wo see their power being undermined by the rise of large-scale, Google-LinkedIn, providers, who’s income, corporate relationships and global reach will shift the power balance in the industry.

Given this awareness of the risks of change, it is beholden on the new technology firms to be sensitive to the needs of all stakeholders. We advocate a Green Ocean strategy (Passmore and Mir, 2020). Under such a strategy, the focus is on collaboration, seeking sustainable win-win outcomes, which benefit all stakeholders, and take at their heart environmental considerations and ethical management, balancing such needs against the drive for quarterly revenues.

## The Implications for Positive Psychology - Coaching Research

In previous papers, we have proposed a model reviewing the journey of positive psychology coaching research (Passmore and Fillery-Travis, 2011). This offered a series of broad phases, noting the journey of published papers from case studies to more scientific methods, such as randomised control trials, between the 1980s and 2010. The past decade, 2011–21, has witnessed a continued development along the scientific pathway, thanks to the work of researchers such as Anthony Grant, Rebecca Jones, Erik de Haan and Carsten Schermuly.

Specifically, the publication of randomised control trials has grown from a handful of papers in 2011 to several dozen by 2021, while still limited in comparison to areas of practice such as motivational interviewing (Passmore and Leach, 2021), the expanded data set has provided evidence for systematic literature reviews and combination studies, such as meta-analysis. These papers have provided evidence that coaching works, with an effect size broadly similar to other organisational interventions, as well as giving insights at to the most important ingredients of the coaching process.

It is this blossoming of higher quality, quantitative studies, which has led us to believe the science in coaching is maturing. While much work still needs to be done over the coming decade, the insights to date can be used to inform practice at a scale leading to Stage 4 in our 5P coaching sector model.

The coming decade may see opportunities for greater collaboration between coach service providers, as these organisations increase in scale and profitability, and university researchers, keen to access large data sets enabled by the greater use of technology and the global scale of the new coach service providers.

Reflecting these industry changes and the proliferation of research, we have also updated the research journey model, reflecting these developments. We suggested the emergence of new phase of research exploring individual, exceptions and negative effects of coaching (Passmore, 2016; Passmore and Theeboom, 2016). This has started to happen with work by Schermuly and Grabmann (2018) and De Hann (2021). We have linked research papers to the model of coach development in Table 4 and have extended it to create 10 phases.

**Table 4:** 10-phase model of coaching research.

Phases	Examples of study
Phase 0 - Pre-science	Trueblood, 1911; Huston, 1924; Gordy, 1937; Bigelow, 1938
Phase 1: Case study and surveys	Diedrich, 1996; Winum, 2005
Phase 2: Qualitative studies – theory generation	Duff and Passmore, 2010
Phase 3: Small sample RCT’s and theory testing	Grant et al., 2010
Phase 4: Large sample RCT’s	Passmore and Rehman, 2010
Phase 5: Meta-Analysis studies	Jones et al., 2016; Theeboom et al., 2014; Sonesh et al., 2015
Phase 6: Systematic Literature Review	Grover and Furnham, 2016; Athanasopoulou and Dopson, 2018; Bozer and Jones, 2018
Phase 7: Identifying the active ingredients	De Haan, 2021
Phase 8: Exploring difference and exceptions	Schermuly and Grabmann (2018); De Hann, 2021
Phase 9: The coaching assignment	Research questions might include: How does homework impact on outcomes across the coaching assignment? How does a tripartite commissioning, review and evaluation impact on coaching outcomes?
Phase 10: The System	How does coaching impact on the wider system of stakeholders? How does team coaching differ in its relationship, active agreements and outcomes from 1–1 coaching?

The emergence of large coaching providers, operating on digital platforms, with the ability to collect, hold and analyses large volumes of data, the opportunity exists to significantly step up the quantity and quality of research including RCT’s and exploring exceptions and specific presenting issues, ingredients and tools. Of specific interest will be questions including: How does the coach (or client) personality impact on the relationship and outcomes? What roles does similarity in terms of race, gender or sector background have on outcomes? What factors contribute to client trust? How significant is empathy as a factor and in want types of coaching is it most valued? What role do discovery meetings, contracting, external support networks and ‘homework’ play in successful coaching assignments?

Over the next 20years, we can start to unpick these aspects with the help of big data, and unlike some aspects of technological research, let us argue in favour now of sharing knowledge through Open Access, so everyone can gain, and the quality of each and every coaching conversation can be enhanced.

## Conclusion

In this paper, we have explored coaching as an expression of positive psychology. We have offered two conceptual frameworks, one for research and one for practice. We hope these frameworks will stimulate further discussion by coaching and positive psychology communities. Our view is that the coaching has become an ‘industry’ and is following a pathway of development similar to many other industries. Recent technological developments, combined with a quickening pace in coaching research, will move coaching from a ‘cottage industry’ towards a fully mechanised process, enhancing accessibility, consistency and reducing cost. This will start with platforms and is likely to lead towards a growing use of automation. This scale provides opportunities for more data, more research and a deeper understanding of the intervention, creating a virtuous circle of development. This too will stimulate the continued development of coaching research pathways considering the assignment and the wider system.

## Data Availability Statement

The original contributions presented in the study are included in the article, and further inquiries can be directed to the corresponding author.

## Author Contributions

All authors listed have made a substantial, direct and intellectual contribution to the work and approved it for publication.

## Conflict of Interest

Authors JP and REK were employed by company CoachHub GmbH.

## Publisher’s Note

All claims expressed in this article are solely those of the authors and do not necessarily represent those of their affiliated organizations, or those of the publisher, the editors and the reviewers. Any product that may be evaluated in this article, or claim that may be made by its manufacturer, is not guaranteed or endorsed by the publisher.
